# Knox County Medical Society

**Published:** 1854-01

**Authors:** J. W. Spalding, L. C. Lane

**Affiliations:** Pres’t.; Henderson; Sec.; Henderson


*Knox County Medical Society.*

In accordance with previous notice, Knox Co. Medical Society met on the 9th instant, at Henderson.

The rcll being called, four of the members were absent. The case of those absent at the preceding meeting being next taken up, the causes of non-attendance which were offered, were deemed sufficient grounds for absence.

On invitation of the society, Dr. J. L. Fifield of Victoria, became a member.

On recommendation of the president the following committees were appointed, with orders to prepare written reports upon the subject assigned to them; to be read at the next meeting of the society, viz. : Drs. Duncan, Edgerton and Spalding, to report on the epidemics most prevalent in the vicinity; Drs. Lane and Fifield, to report upon the state of the profession in the county; Drs. Cooper and Brewer, to report upon the subject of quackery. The society then entered into a spirited discussion relative to the propriety of striking a line of demarcation between the members of this society, and those members of the profession in the vicinity who refuse to attach themselves to it. It was finally left to the option of the committee on the " state of the profession in the vicinity, " to report on this subject, as they might see fit. The society next proceeded to a report of cases. Allusion was made to the recent dysenteric epidemic which has prevailed, during the last season at Berwick. Dr. Spalding reported the phenomena, which were present in the disease as witnessed by himself. The disease seems in a few cases to have supervened upon measles, yet in the majority of instances, appears to have been idiopathic in character. The attention of the society was next directed to the treatment of dysentery in general.

On motion, the society instructed the secretary to forward a copy of the proceedings of this meeting for publication in the *Knoxville Journal* and in the *North Western Med. and Surg. Journal.*

On motion, the society adjourned to meet again at Galesburg, at its next quarterly meeting.

J. W. SPALDING, M.D., Pres't.

L. C. LANE, M.D., Sec.

HENDERSON, Oct. 9th, 1853.

